# Phytomers, collet and founder cells: a “universal” plant embryonic body plan from a developmental, molecular, and evolutionary perspective

**DOI:** 10.3389/fpls.2025.1521527

**Published:** 2025-08-13

**Authors:** Prakash Venglat, Perumal Vijayan, Timothy F. Sharbel, Abidur Rahman, Karen Tanino

**Affiliations:** ^1^ College of Agriculture and Bioresources, University of Saskatchewan, Saskatoon, SK, Canada; ^2^ Department of Plant Biosciences, Faculty of Agriculture, Iwate University, Morioka, Iwate, Japan

**Keywords:** embryo - evolution, model, development, embryophytes, GRN

## Abstract

This article presents a novel perspective on plant embryogenesis, fundamentally differentiating it from the animal embryo model upon which plant models have long been based to discern the genetic and molecular mechanisms. We propose a plant embryonic body plan that aligns developmental and evolutionary insights across all five embryophyte groups (bryophytes, lycophytes, monilophytes, gymnosperms, and angiosperms). This conceptual model is grounded in a Reprogramming Potential (RP) involving an activation (RP1+) -suppression (RP1-) switch (RP1+/RP1-), which integrates embryonic development in a stepwise manner across diverse embryophytes. We further explore the evolutionary trajectory of this body plan, tracing the gradual assembly of the embryophyte genetic toolkit from bryophytes to angiosperms. Key developmental processes, such as the emergence of shoot and root meristems, vascular tissues, and seeds, are also examined within an evo-devo framework. Plant phenotypic plasticity, fundamental to their adaptation and survival, is manifested in two key hallmarks: (A) the iterative, modular growth of shoot and root units, and (B) their remarkable regenerative potential. While traditionally viewed as separate phenomena, we propose a novel, integrative model that connects these hallmarks within the context of plant embryogenesis. Our “proposed universal plant embryonic body plan” reconciles the genetic and molecular mechanisms of *Arabidopsis thaliana* embryogenesis with the contrasting developmental patterns observed in monocots. This unified model also integrates the concept of root founder cells and collet (shoot-root junction) into an embryonic framework facilitating the study of gene regulatory networks that underpin root evolution and its architecture.

## Introduction

Phenotypic plasticity is at the core of the ability of plants to adapt to their changing environments, fundamental to their ability to survive and thrive. In majority of the land plants, the body plan is elaborated post-embryonically in an iterative manner by the shoot and root meristems and its associated procambial tissue (Rensing and Weijers, 2021). The iterative units of the shoot are referred to as phytomers, and the root forms a branching architecture by an iterative patterning of the lateral roots on the primary root, with initiation of the shoot-borne and adventitious roots depending on the species (Perianez-Rodriguez et al., 2014). The other important basis for phenotypic plasticity observed in plants is the high degree of regenerative capacity as witnessed during: (a) formation of asexual propagules coupled with dormancy, (b) vegetative propagation, (c) wound healing and (d) reprogramming of tissue explants in tissue culture to form callus, somatic embryos, shoot and root meristems (Ikeuchi et al., 2019; Mathew and Prasad, 2021). These two hallmarks of plants, i.e., (A) the iterative and adaptive body plan (Hallmark 1 [H1]) and (B) phenotypic plasticity defined by their reprogramming/regenerative potential (RP) (Hallmark 2 [H2]), differentiate them from the animal body plan and development. By contrast, the animal body plan is mostly embryonically determined and shows lesser regenerative capacity and phenotypic plasticity compared to the plants. In this article, we bring forth a “plant-specific” embryonic framework integrating the evolutionary and developmental trajectories of land plant embryogenesis, bringing forth a universal self-organizing logic for plant development and regeneration. This conceptual model also integrates the two hallmarks from a shoot – root perspective, the fates of which are reprogrammable during regeneration. A deeper understanding of how plant phenotypic plasticity is established during embryogenesis is crucial as this knowledge will help develop improved models that explain the modular and adaptable nature of plant growth. Ultimately, this will facilitate designing the crops that will be better equipped to withstand increasingly stressful climates.

To propose a new framework for plant embryogenesis, we first critique the limitations of conventional paradigms derived from animal development. We then trace the evolutionary assembly of the embryophyte genetic toolkit, examining key developmental innovations from bryophytes to angiosperms. Next, we demonstrate the need for a more inclusive model by showing the contrasting and distinct developmental patterns seen in the dicot model *Arabidopsis thaliana* (hereafter referred to as Arabidopsis) model and monocots. Finally, we propose a “universal” model hypothesis for plant embryogenesis that integrates these developmental, molecular, and evolutionary insights to explain the modular and regenerative nature of plant life.

## Contrasting developmental paradigms: the basis for two plant hallmarks

**Figure 1:**
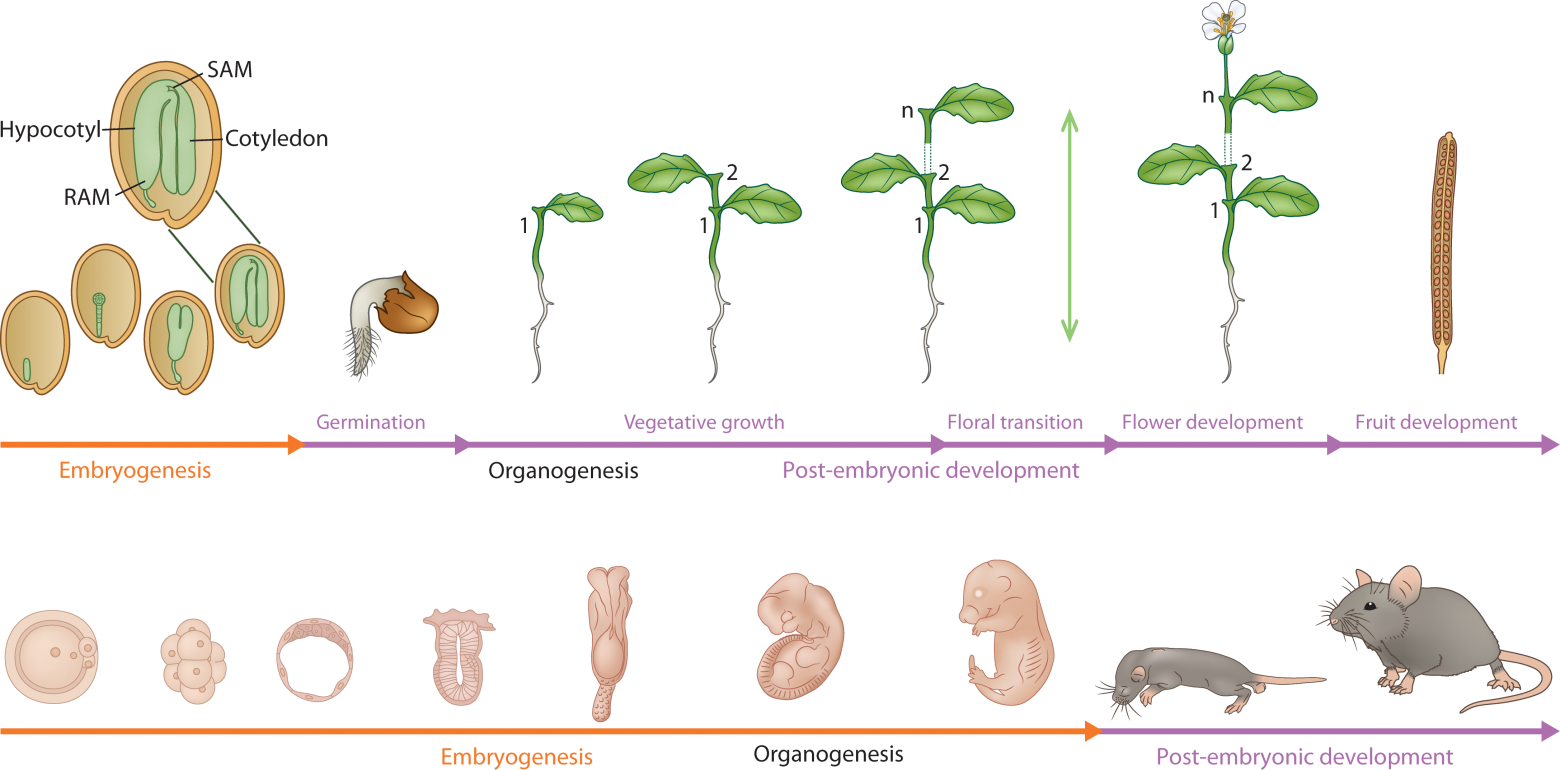
A comparative developmental profile of embryonic (orange line) and post-embryonic development (purple line) in a plant (mustard plant) and a mammal(mouse). The figure captures the fundamental difference between plant and animal embryogenesis. The organs are iteratively produced post-embryonically in plants by the shoot and root meristems, whereas the organs are formed (organogenesis) during embryogenesis in animals. The green vertical double-ended arrow indicates indeterminate growth. Embryonic phase in plants establishes the embryonic body plan (zoomed image) with shoot (SAM)/root (RAM) meristems and embryonic organs (cotyledon, hypocotyl).

Both plants (sporophyte phase) and animals begin their lifecycle with the formation of the zygote, which is totipotent, the capacity to give rise to the whole organism (Baroux et al., 2008). The genetic model of plant embryogenesis is based on mutants isolated from Arabidopsis but began with a template that was developed based on an animal model, Drosophila (Mayer et al., 1991). Compared to the stem cell niches located in specific organs in animals, during the process of plant embryo development from the zygote, polarity and patterning of the body axis and differentiation of cell/tissue types result in the segregation of the pluripotent stem cells to specific differentiation programs, i.e., the shoot/root meristems and procambium in plants (Scheres, 2007). Thus, the pluripotent stem cell programs that are assembled during embryo development from a totipotent zygote are wired differently to their respective differentiation programs in plants and animals (Heidstra and Sabatini, 2014). This gives rise to an open indeterminate type of post-embryonic development in plants as opposed to a determinate organismal growth, development, and homeostasis in animals. An illustration comparing the life cycle of a plant, and an animal further shows the fundamental difference in their developmental programs (**Figure 1**). Plant embryogenesis assembles the meristems that participate in iterative and sequential organ growth during post-embryonic development whereas in animals, embryogenesis forms all the adult organs that undergo further growth during post-embryonic development. (Beyond Animal Blueprints: The Plant Developmental Paradigm; detailed in **Box 1**)

Box 1 **Beyond animal blueprints: the plant developmental paradigm.**

We suggest that while much of our understanding of plant embryogenesis has been achieved through the lens of animal development, the fact that plants are fundamentally distinct with reference to their architectural and regenerative principles provides an opportunity for discovery. While animals largely establish their complete body plan during embryogenesis, plants forge a foundational toolkit—the meristems—to build and rebuild themselves continuously throughout their lives. This distinction, rooted in a unique evolutionary history and managed by sophisticated molecular circuitry, necessitates a shift to a plant-specific framework.

**Continuous construction vs. a fixed body plan**

Animals

Development is largely determinate. The embryonic phase involves complex cell migrations to create a body plan where all major organs are formed early in the life cycle. While lineage does play a significant role in determining cell fate in animals, the determinate nature of their body plan formation during embryogenesis, and the post-embryonic maintenance of organs with stem cell niches involves positional information, induction, and cell-cell communication.

Plants

Embryogenesis establishes self-renewing stem cell niches (meristems) that act as engines for lifelong, indeterminate growth. This allows for the iterative, modular production of new organs (phytomers), which we term Hallmark 1. Because rigid cell walls prevent cell migration, a plant cell’s destiny is determined not by its ancestry but by its position and the signals it, for the most part, receives from its neighbors.

**Unparalleled regenerative potential**

Plants possess an extraordinary ability to reprogram their developmental fate, a feature we define as Hallmark 2. Differentiated somatic cells can change their fate in response to internal or external cues to regenerate new organs or individuals - a capacity for renewal far exceeding that of most animals.

**The logic of molecular switches**

Underlying this developmental plasticity is a distinct logic encoded in Gene Regulatory Networks (GRNs). These networks function as dynamic activation-suppression “switches” that govern cell fate. For example, the fundamental decision to become a shoot or a root is controlled by an antagonistic relationship between key transcription factors that mutually repress one another. These molecular switches are the engines of reprogramming (Hallmark 2), allowing cells to adopt or suppress developmental fates based on positional information and environmental triggers.

**A proposed integrated model**

We propose an integrated model as a basis for understanding the diverse developmental strategies that exist across the plant kingdom, especially the contrasting patterns seen in monocots. Considering plant-specific principles, it is hypothesized that the embryonic origin of meristems with lifelong iterative function (Hallmark 1) in conjunction with a profound regenerative capacity (Hallmark 2), provide the building blocks for GRNs to evolve modular responses to selection pressure.

The developmental hourglass model emerged out of comparative embryological studies in animals, the narrow part of the hourglass referred to as the phylotypic stage, the least divergent stage during their ontogenies (Irie and Kuratani, 2014). The phylotypic stage represents the highly conserved stage during body plan establishment in the diverse animal lineages. This was further reflected in the gene expression studies of animal embryo development using phylotranscriptomic analysis, which showed a transcriptomic hourglass with the narrow part of hourglass representing the transcripts of least divergent and most conserved genes (Irie and Kuratani, 2011) which function in the establishment of embryonic body plan in diverse animal species (Richardson, 2012). Phylotypic stage represents the empirically viable modern version of the “biogenetic law” – ontogeny recapitulates phylogeny (Niklas et al., 2016). Similar work using the transcriptomes of Arabidopsis and wheat embryo stages representing an ontogenetic sequence showed that the transcriptomic hourglass model is also observed during plant embryogenesis in both dicot and monocot species (Quint et al., 2012; Xiang et al., 2019). However, further studies in Arabidopsis showed that the transcriptomic hourglass is also observed during the post-embryonic stages of development, i.e., seed germination (embryo to vegetative stages) and floral transition (vegetative to reproductive stages) reflective of conserved transcriptomes that defines the organization checkpoints enabling a switch between major developmental programs (Drost et al., 2016). This further distinguishes plant and animal development at the molecular level and highlights the open, iterative post-embryonic development in plants (Hallmark 1 - H1) which begins with the establishment of the embryonic shoot program.

Prior to the emergence of the embryophytes, gamete fusion led to a zygote that immediately underwent meiosis to start the haploid gametophyte generation. The embryophyte genetic toolkit which became progressively assembled during the evolution of embryogenesis in the five embryophyte groups has its deep origins in the charophycean algal genome (Bowman, 2022). In plants, the reprogramming (Hallmark 2 – H2) and co-option of developmental and signaling pathways paved the way for the assembly of the genetic programs that regulate embryogenesis and seed development in gymnosperms and angiosperms, notably distinct from animal evolution in which lineage based fating during embryogenesis results in organogenesis and assembly of the body plan (Blanpain and Simons, 2013).

## Assembly of the plant embryophyte genetic toolkit

With the advent of genome sequencing of algae/early land plants and the understanding of genetic and developmental mechanisms, the possibility of emergence of new models provides a rich resource to understand plant embryogenesis (Szövényi et al., 2021). From an evolutionary perspective, the embryophytes, comprising of bryophytes, lycophytes, monilophytes, gymnosperms and angiosperms showed: decreasing lifespan of the gametophytes, increasing degrees of complexity of their embryonic body plan and the increasingly prolonged lifespan of sporophytes they form (Szövényi et al., 2019).

Characteristic of eukaryotes is a life cycle defined by an alternation between haploid (gametophyte) and diploid (sporophyte) phases, initiated by meiosis and gamete fusion, respectively. In multiple eukaryotic lineages, including embryophytes, a genetic switch based on expression of paralogous homeodomain (HD) proteins in the two gametes directs the haploid to diploid transition in gene expression. In the chlorophyte alga *Chlamydomonas*, the haploid to diploid transition is mediated by a heterodimer formed between a minus-gamete-expressed KNOX TALE-HD protein and a plus-gamete-expressed TALE-HD BELL protein following gamete fusion (Hisanaga et al., 2021). Some of the early zygotic targets of the KNOX-BELL heterodimer encode enzymes that remodel the cell wall, preparing the zygote to become a dormant dispersal agent (Bowman, 2022). A similar, although modified, system operates in mosses and liverworts, with *KNOX* genes expressed in the egg cell and *BELL* genes expressed in both sperm and egg cells (Bowman et al., 2016). *Marchantia MpKNOX1* and *MpBELL* are also expressed in proliferating tissues in developing sporophytes, suggesting similarity with KNOX1 functions in angiosperm meristems that are formed during embryo development. Both KNOX1-BELL and KNOX2-BELL heterodimers have been retained and co-opted into developmental patterning roles throughout the angiosperm diploid sporophyte development (Bowman, 2022).

Several features that give rise to the embryonic body plan from bryophytes to angiosperms, include: (i) early evolution of indeterminacy [e.g., hornwort sporophyte], (ii) evolution of the shoot meristem and its sustained ability to maintain growth, (iii) evolution of the procambium and the vascular initials, (iv) evolution of the root meristem and (v) evolution of the cotyledon and the seed. These developmental milestones were eventually integrated during evolution by divergent and convergent mechanisms to form the bipolar embryo seen in seed plants.

## Evolutionary origins of the sporophyte body plan: building the shoot and root meristems

The emergence of the building blocks that paved the way for the formation of the meristems and an embryonic body plan in later land plants began with the establishment of the bryophytes as the first land plants with a sporophyte that has a distinct morphology.

### Sporophyte body plan and emergence of shoot meristem

The sporophyte that develops from the zygote in bryophytes (liverwort, hornwort, moss) is a spore bearing sporangium with limited growth. In *Physcomitrium patens*, a moss model (hereafter referred to as *Physcomitrium*), the developing sporophyte shows distinct stages: apical cell divisions, merophyte divisions, merophyte division following cessation of apical cell activity, proliferative activity of the intercalary region, swelling sporangia and its development to maturity (Coudert et al., 2019). Mutation in a KNOX class I (KNOX1) homolog *mkn2*, resulted in the absence of proliferative activity in the intercalary region whereas rest of the sporangium developed normally resulting in a stunted sporangiophore. The MKN2 was further shown to activate *PpIPT3* which activates cytokinin biosynthesis, implicating a role for the KNOX-cytokinin pathway in the intercalary proliferative activity (Coudert et al., 2019). The KNOX-cytokinin pathway plays an important role in angiosperm shoot meristem initiation and maintenance (Hay and Tsiantis, 2010) and its ancestral role in bryophytes hints at its fundamental role in the emergence of stem cell systems (Coudert et al., 2019). Diversification via duplication of the KNOX family into two classes in bryophytes resulted in new functions for KNOX class II (KNOX2) genes. Deletion of two KNOX2 genes in *Physcomitrium* resulted in gametophyte development from diploid embryos in the absence of meiosis. In this context, KNOX2 genes have evolved a repressive role, preventing the gametophyte-specific body plan from developing the sporophytic phase (Sakakibara et al., 2013).

The terminal nature of the sporophyte is defined by the formation of a single sporangium in bryophytes (e.g., *Physcomitrium*). Genetic pathways that shift the determinate nature of the sporophyte were under selection pressure due to the habitat shifts in the environment, as gleaned from the geology of the Rhynie chert and the well-preserved fossils in that region (Garwood et al., 2020). This likely paved the way for the emergence of the stem cells of the shoot meristem. Characterization of the sporophyte-specific gene expression in *Physcomitrium* identified sporophytic-specific transcription factors, specifically those encoded by *TCP* genes that are known to repress branching in angiosperms (Martín-Trillo and Cubas, 2010; Ortiz-Ramírez et al., 2016). Deletion of *PpTCP5* resulted in supernumerary sporangia attached to a single seta, indicating an ancestral role for *TCP* genes and sporophyte morphology in *Physcomitrium* (Ortiz-Ramírez et al., 2016).

The origin of auxin’s role in the elaboration of the sporophyte form is a pertinent question to address with reference to emergence of the shoot meristem in land plants. PIN proteins play a foundational role in the transport of auxin, setting up a template for auxin driven morphogenesis. The role of auxin in the bryophyte sporophyte and during gametophyte development was studied in *Physcomitrium* by characterizing *PpPIN* genes (Langdale, 2014). PIN based polar auxin transport was shown to play a critical role in the tip growth of protonemal cells and further elaboration of the gametophyte, thereby ascertaining the role of polar auxin transport in morphogenesis (Bennett et al., 2014; Viaene et al., 2014). Furthermore, *pin* mutants of *Physcomitrium* occasionally formed branched sporophytes confirming a role for auxin in the elaboration of the sporophyte body plan in bryophytes (Bennett et al., 2014).

The fossil record of extinct land plants that fall between the extant species of bryophytes and lycophytes reveal several attempts at forming sporangiophores that branched sympodially (bifurcating branching pattern) to give rise to two or more sporangia. Earlier forms had few sympodial branches with sporangia at their tips (i.e., *Partitatheca*) whereas the later forms of fossilized tracheophytes displayed elaborate rhizomatous growth and multiple sympodial branching with vascularization (i.e., *Aglaophyton, Cooksonia, Rhynia*). Progressive emergence of the multicellular apical shoot meristem from simpler stem cell programs, e.g., the intercalary seta cells of bryophytes and the apical initials of lycophytes, is one hypothesis actively being pursued (Fouracre and Harrison, 2022). Amongst the fossilized tracheophytes, the lateral branching of the sporophytes and elaboration of its vasculature led to larger forms (e.g., *Asteroxylon*, *Lepidodendron*, *Paracalamitina*, *Archaeopteris*) along with the first sign of root development in land plants (Harrison and Morris, 2018).

### Emergence of the root meristem

Observation of extinct and extant early land plants reveal that the root meristem emerged independently in lycophytes and euphyllophytes (monilophytes, gymnosperms and angiosperms) (Hetherington and Dolan, 2019; Spencer et al., 2021). Prior to the emergence of the root meristem, the unicellular rhizoid-based systems anchored the shoots in bryophytes (Kenrick and Strullu-Derrien, 2014). Roots evolved gradually and independently in several clades over ~50 million years (Devonian period – 416 to 360 million years). Along with the elaboration of early rooting systems, the vasculature of extinct land plants reveal an elaboration of the vascular system, cambial activity (emergence of another meristem), secondary growth and the formation of wood (Spencer et al., 2021).

The fossil records of predicted common ancestors of lycophytes and euphyllophytes preserved in the Rhynie chert (e.g. *Horneophyton, Aglaophyton, Rhynia, Nothia* sp.) reveal non-vascular and vascular plants lacking root systems (Hetherington and Dolan, 2019). The presence of roots in extant lycophyte and euphyllophyte lineages indicates that there were at least two independent origins of roots among extant vascular plants. The early root meristems formed in the extinct and extant lycophytes exhibited determinate growth ending up bifurcating into two meristems typical of sympodial branching roots (Hetherington and Dolan, 2019). Careful examination of the fossilized root meristems of *Asteroxylon mackiei* revealed that they lack root caps compared to those formed later during evolution (Hetherington and Dolan, 2018). This supported the independent origin of root meristems in the lycophyte and euphyllophyte lineages and highlights the stepwise evolution of root meristem with the earlier formed root meristems lacking root caps and the later formed root meristems developing root caps. A detailed view of the relationships of the early land plant fossils and their placement in the various land plant groups is given in (Crane et al., 2004) and summarized in **Box 2**.

Box 2 **Fossils and land plant phylogeny.**

**Figure:**
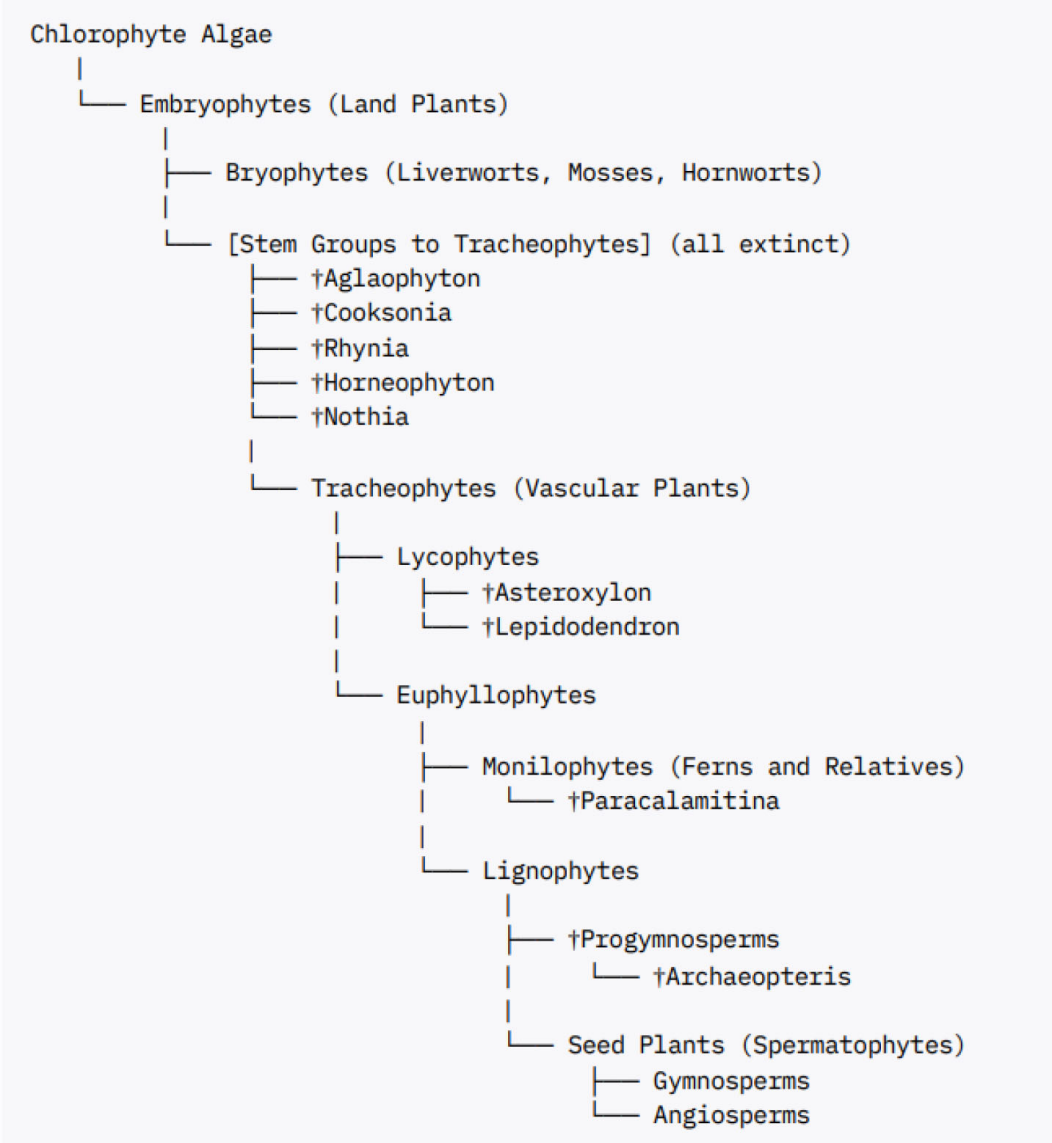

Notes:

The dagger (†) denotes extinct groups.

All placement reflects the best-supported current understanding of fossil and living plant relationships.

### Dicot vs monocot embryogenesis

All the crop species grown for nutritious seeds/grains belong to the dicot and the monocot groups of angiosperms, whose seeds are characterized by the embryo and/or the endosperm contained with a seed coat (Venglat et al., 2014; Armenta-Medina et al., 2021). Arabidopsis has been used extensively to characterize patterning events during embryogenesis, and their corresponding genetic and molecular mechanisms (Palovaara et al., 2016). The current embryonic body plan model in angiosperms, which is largely based on genetic and molecular studies in Arabidopsis, defines: (1) apical-basal polarity, (2) embryo vs suspensor identity, (3) bilateral patterning to form the two cotyledon primordia and, radial patterning leading to the three major tissue types, i.e., protoderm, ground tissue and vascular tissue, and (4) formation of the embryonic stem cell niches, shoot apical meristem (SAM) and root apical meristem (RAM) (Palovaara et al., 2016). Embedded within the logic of this embryo model is a bipolar stem cell state of shoot and root stem cell niches with procambial initials placed along the apical-basal axis that gives rise to vascular tissues (xylem and phloem), providing the connectivity to the tissues produced by the SAM and RAM during post-embryonic development. The procambium that is post-embryonically transformed into the bifacial vascular cambium (stem cell niche) contributes to the secondary vascular tissues. Auxin, with its regulated movement and signaling, has a central role in this model as a morphogen which integrates (1) formation an apical-basal axis, (2) bilateral and radial patterning, and (3) specification of the root meristem (Friml et al., 2003).

**Figure 2:**
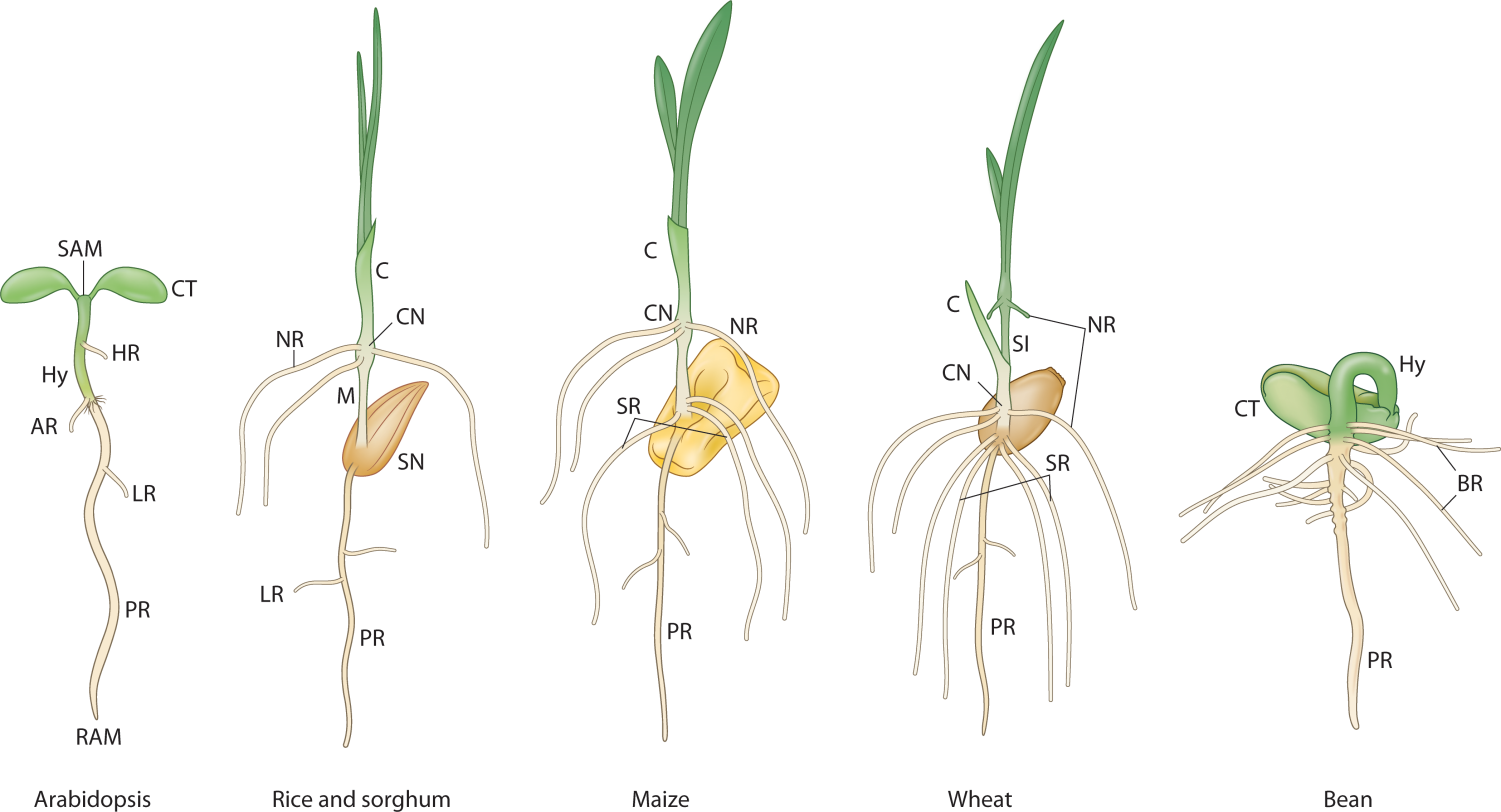
The developing root system during embryonic to seedling transition in different plant species. PR, primary root; AR, anchor root; Hy, Hypocotyl; HR, hypocotyl root; NR, nodal root (embryonic crown root); SR, seminal root; LR, lateral root; BR, basal root; SN, scutellar node; CN, coleoptilar node; SI, sub-crown internode; M, mesocotyl; C, coleoptile; CT, cotyledon; SAM, shoot apical meristem; RAM, root apical meristem; MZ, meristematic zone; TZ, transition zone; DZ, differentiation zone. Adapted from (Salvi, 2017).

However, this Arabidopsis based dicot model is less compatible with the patterning events observed during monocot embryogenesis (e.g. rice, maize, *Brachypodium* and wheat) (Xiang et al., 2019; Hao et al., 2021) which is characterized by a laterally placed shoot meristem, embryonic root meristem initiation in a lateral position in the middle of the embryo, and altered cotyledon primordium initiation, structure and function (**Figure 2**). New organs, including the coleoptile, coleorhiza, and the epiblast, are formed from the intercalary cell division zones. The genetic and molecular mechanisms that unfold during monocot embryo development, including the role of auxin, are less understood (Armenta-Medina et al., 2021). One prominent difference seen in the mature embryos of many monocot species is the number of leaf primordia and embryonic root primordia that emerge during germination and early seedling growth (e.g. 5 seminal root primordia in wheat (Shorinola et al., 2019), 9 in barley, 4 in maize (Avery, 1930)). Even in rice, where a single radicle is prominent in the mature embryo, the rudimentary coleoptile node associated root primordia that emerge during early seedling growth are embryonic in origin (Orman-Ligeza et al., 2013; Hochholdinger et al., 2018). These point to the plastic nature of assembly of the root program during embryo development in monocots. This plasticity is further supported by the idea of genomic ecosystems in the above and below ground plant tissues, which can be differentially invaded by genomic parasites in the same individual, resulting in root-specific elimination of the *Aegilops* sp*eltoides* supernumerary B chromosomes during embryo development (Ruban et al., 2020). The whorls of basal roots that emerge within 3 days of germination at the hypocotyl-root junction in common bean is an example of dicot species showing plasticity in the embryonic-seedling root program (Basu et al., 2007) (**Figure 2**). Regardless of the differences observed in the cell division patterns and morphogenesis during embryogenesis in dicots and monocots, seedlings represent the final functional stage of the embryonic organs with stereotypical positioning of the shoot and root meristems, the cotyledons and the junction (collet) between the shoot and the root (Ten Hove et al., 2015).

## A “universal” model hypothesis for embryogenesis in land plants

Four directions of research that converge on a more universal model hypothesis for embryogenesis in land plants are as follows: (1) Developmental and genetic models of early land plants (bryophyte, lycophyte and monilophyte models) and the evolution of developmental modules, i.e., shoot apical meristem, root apical meristem, cambium (vascular), embryogenesis, shoot and root patterning (Radoeva et al., 2019b); (2) Further refinement of developmental and molecular mechanisms in Arabidopsis embryogenesis and its correlations with monocot (*Poaceae*) embryogenesis (Xiang et al., 2019; Armenta-Medina et al., 2021; Hao et al., 2021); (3) Plant developmental plasticity as gleaned from the molecular mechanisms that regulate plant regeneration and wound healing (Ikeuchi et al., 2019; Mathew and Prasad, 2021); and (4) Contrasting plant versus animal embryo development.

These converge on the two hallmarks (e.g., shoot development as iterative units, root branching elaboration and the reprogramming potential of plants can be used to test the following hypotheses:

1. the bipolar embryonic program with reference to the shoot and the root meristems in seed plants was assembled as modules during early evolution;
2. root originated by a reprogramming event of the shoot.

In the context of our model, a “module” refers to a discrete, functionally integrated unit within the developing embryo. These modules are characterized by a specific set of genes, regulatory interactions, and resulting cellular behaviors that contribute to a distinct developmental process. For example, a module might encompass the genes and pathways responsible for establishing apical-basal polarity, or those governing cotyledon formation.

**Figure 3:**
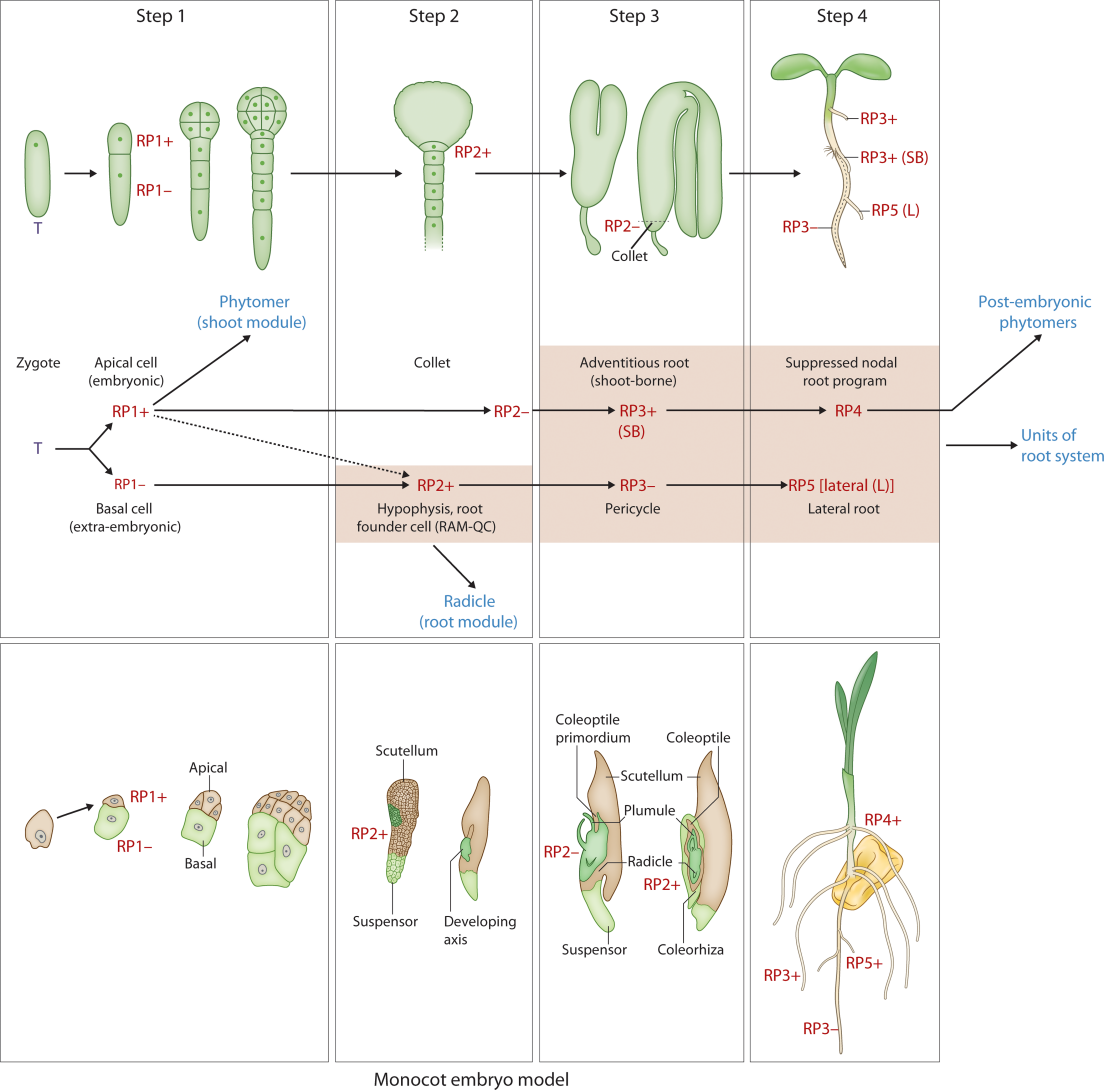
Developmental map of Arabidopsis and monocot embryo development and post-embryonic development with reference to the two hallmarks (iterative, regenerative) Upper panel: Step 1 - zygote to two-cell stage; Step 2 - hypophyseal cell specification (32-cell stage); Step 3 – the collet-hypocotyl partitioning separating the phytomer from the radicle; Step 4 – phytomer bilateral symmetry; Lower panel: RP1+ - embryonic potential; RP1- - suppressed embryonic potential; RP2+ - activation of the root program; RP2- - suppressed shoot-borne root program (in the collet region); RP3+ - activation of the shoot-borne root program; RP3- - suppression of lateral root program (pericycle cell fate); RP4 – suppressed nodal root program; RP5 – activated lateral root program. T, totipotency; R, re-generative potential; (+) activated; (-) suppressed; SB, shoot-borne; L, lateral. While the upper panel of image 3 illustrates the Arabidopsis (dicot) pattern, the lower panel depicts a simplified monocot representation. Specifically, the lateral positioning of shoot and root meristems in monocots, relative to surrounding embryonic and maternal tissues, can be understood within our framework. Step 1, establishing polarity, is evident in the initial asymmetric division. Step 2, tissue specification, accounts for the differentiation leading to the distinct positioning of these meristems. Step 3, organogenesis, reflects the subsequent development of these lateral meristems within the monocot embryo’s unique architecture. Finally, regarding Step 4, the “universally conserved” aspect refers to the establishment of the basic body plan, not the precise form of cotyledon symmetry. Monocots, while lacking symmetrical cotyledons, still undergo a defined phase of embryo maturation and growth, aligning with the core concept of Step 4.

**Figure 4:**
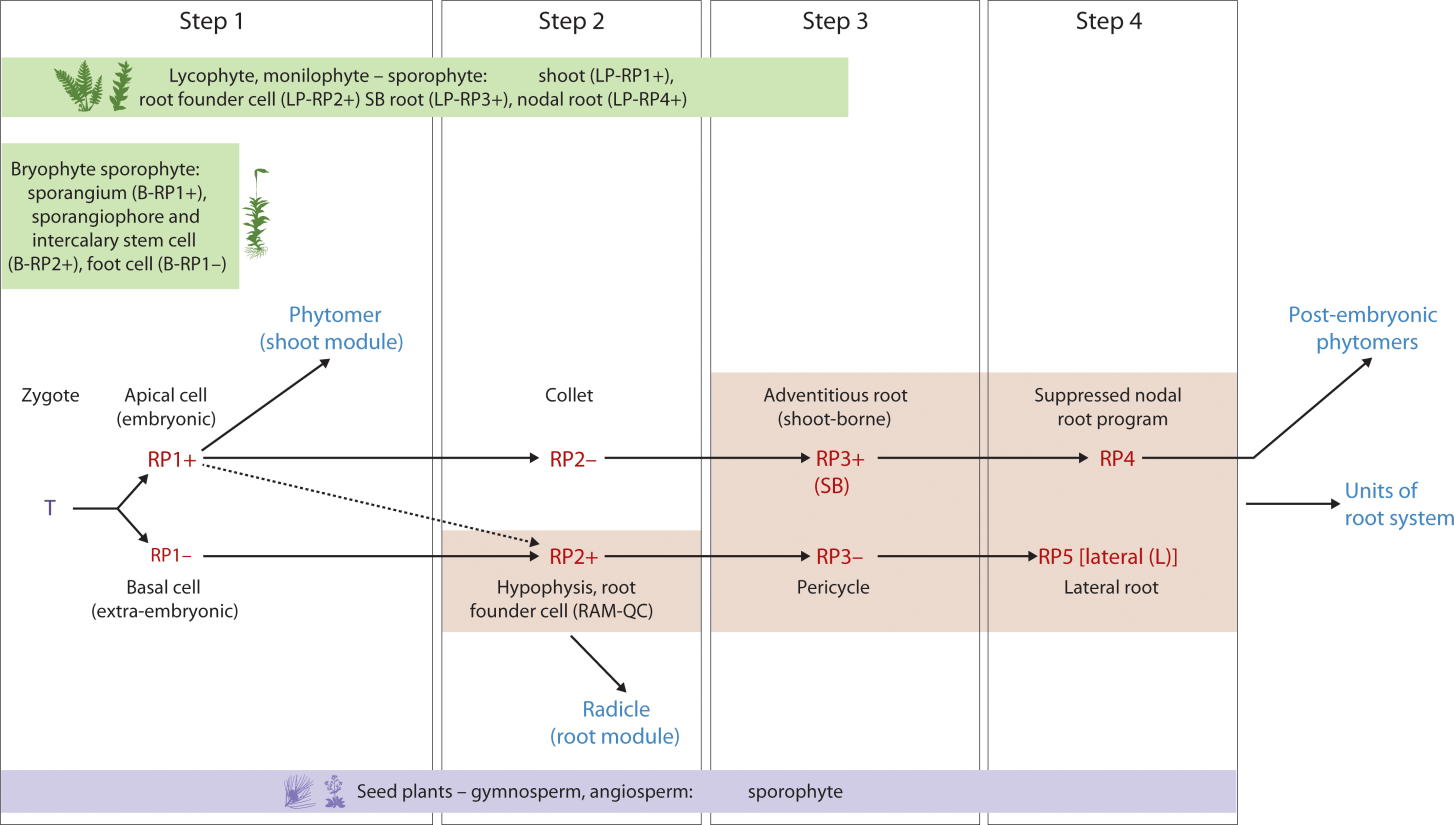
Hypothetical Evo-Devo map of embryo development and post-embryonic development with reference to the two hallmarks (iterative, regenerative) in seed plants (e.g., Arabidopsis) and the corresponding stages in bryophytes, lycophytes and monilophytes. Upper panel: Step 1 - zygote to two-cell stage; Step 2 - hypophyseal cell specification (32-cell stage); Step 3 – the collet-hypocotyl partitioning separating the phytomer from the radicle; Step 4 – phytomer bilateral symmetry; Lower panel: RP1+ - embryonic potential; RP1- - repressed embryonic potential; RP2+ - activation of the root program; RP2- - repressed shoot-borne root program; RP3+ - activation of the shoot-borne root program; RP3- - suppression of lateral root program (pericycle cell fate); RP4 – suppressed nodal root program; RP5 – activated lateral root program. T, totipotency; R, re-generative potential; (**+**) activated; (**-**) suppressed; SB, shoot-borne; L, lateral; LP, Lycophyte/monilophyte (Pteridophyte); B, Bryophyte.

To test these hypotheses, the Arabidopsis ontogenetic sequence of embryogenesis will be described in 4 steps, each step defined by the two hallmarks [**Box 3-(I)**]. Some of the underlying mechanisms and cell fates are provided in **Box 3-(II)**. The linkages between these steps and their evolvability can be used to develop an integrative “universal” model hypothesis for plant embryogenesis from a developmental (**Figure 3**) and evolutionary perspective (**Figure 4**). Arabidopsis embryogenesis is well-defined and provides a detailed exemplar to overlay the model’s components while testing the evolutionary assembly of these modules necessitates further studies of comparative functional genomics and developmental studies across embryophyte phylogeny.

Box 3 **Modular ontogeny for the proposed universal embryo model.**

(I) Four modular steps in the ontogenetic sequence of plant embryo development

**Hallmark 1 (H1):** Activation of the embryonic shoot program

**Hallmark 2 (H2):** Reprogramming the basal fate of the embryonic shoot program

**Step 1: zygote**

Hallmark 1 (H1): Apical – basal polarity: embryo vs extra-embryonic (suspensor) identity

Hallmark 2 (H2): Reprogramming the embryonic fate in the suspensor.

**Step 2: 32-cell stage**

H1: Shoot – vascular – root partitioning

H2: reprogramming the shoot/suspensor end; activation of the root program; suppression of shoot fate.

**Step 3: shoot – root partitioning**

H1: hypocotyl – root partitioning

H2: reprogramming the hypocotyl/collet (phytomer) end; activation of the shoot-borne root program; suppression of the SB (shoot-borne) root program.

**Step 4: patterning the embryonic “phytomer”**

H1: embryonic “phytomer” patterning

H2: reprogramming of the embryonic “phytomer”; activation of the adventitious root program; suppression of the adventitious root program

**(II) Activation** – suppression switch defined in each modular step of plant embryo development

**RP** – Reprogramming/Regenerative potential

Step 1 – RP1: inductive interaction (polarity)

**RP1+** = activation of embryonic program; **RP1-** = suppression of embryonic program

Step 2 – RP2: morphogenic gradients (auxin, HD-ZIP III, PLT); primary root program founder cell – hypophysis in Arabidopsis

**RP2+** = activation of the primary root program; **RP2-** = suppression of shoot-borne root program (in the collet region)

Step 3 – RP3: root developmental gradient; founder cells – shoot-borne roots

**RP3+** = activation of the shoot-borne root program; **RP3-** = suppression of lateral root program

Step 4 – RP4: patterning gradient (bilateral – radial patterning), founder cells – adventitious root program

Post-embryonic step – lateral root program (pericycle); founder cells in the phytomer (pericycle-like cells)

**RP4** – suppressed nodal root program; **RP5** – activated lateral root program

### Step 1: embryonic vs extraembryonic cell identity (**Figure 3**)

In a laser ablation study of Arabidopsis embryo development (Liu et al., 2015), embryo ablated from the suspensor at the 8-cell stage resulted in the uppermost suspensor cell taking up the embryonic fate after 3 days in culture. This was also repeatable at the 32-cell stage but not at the heart stage. The presumed mechanism is the upward movement of auxin from the ovule via the suspensor resulting in auxin accumulation in the uppermost suspensor cell. At the heart stage, the suspensor cells most likely have commenced programmed cell death and thus reprogramming was not possible (Liu et al., 2015). This demonstrates the suppressed embryonic state (Reprogramming Potential [RP1-]) of the suspensor cell that has lost contact with the embryonic cell (**Figure 3**) whereby RP1+ denotes the activation of the embryonic program in the apical cell after the first asymmetric division of the zygote (**Figure 3**). Other studies have demonstrated the importance of auxin signaling in suspensor and embryo fate (Radoeva et al., 2019a) and suspensor driven embryogenesis in Arabidopsis (Radoeva et al., 2020).

### Step 2: shoot vs root fate

During Arabidopsis embryo development, the auxin maxima in the uppermost suspensor cell at the 32-cell stage embryo initiates a cascade of events resulting in the hypophyseal cell fate and further development of the root stem cell niche (Scheres et al., 1994; Friml et al., 2003; Aida et al., 2004). The apical half of the embryo is patterned into a bilaterally symmetrical form with a shoot meristem flanked by two cotyledonary primordia (Palovaara et al., 2016; Zhang et al., 2017). The *topless* mutant of Arabidopsis confers a root fate to the shoot (Long et al., 2002, 2006). The TOPLESS (TPL) protein functions as a transcriptional co-repressor in the auxin signaling pathway (Szemenyei et al., 2008). In the apical-basal patterning of the embryo, TOPLESS (TPL) acts as a co-repressor and interacts with auxin-regulated Aux/IAA proteins (Szemenyei et al., 2008). In the apical domain, TPL activity contributes to preventing root fate [by the suppression of PLT activity (Smith and Long, 2010)], while in the basal domain, auxin promotes PLT activity for root specification (Aida et al., 2004). Furthermore, TPL, working in conjunction with PLETHORA (PLT) proteins, is involved in confining the expression of shoot-promoting HD-ZIP III factors to the apical domain, effectively repressing shoot fate in the basal domain (Smith and Long, 2010). Alleviation of the suppression of the HD-ZIP III factors in the basal domain of the embryo resulted in the transformation of the root to shoot (Grigg et al., 2009). This demonstrates the repressed state of shoot fate in the basal domain which when relieved, produces shoots at both ends. Activation of hypophyseal cell fate and root development is referred to as RP2+ (**Figure 3**). The migration of the HD-ZIP III factors into the basal domain, activating a shoot program, is a reversion back to an apical fate RP1+ (**Figure 3**). **Box 4** provides a framework for a gene regulatory network (GRN) representation of the shoot – root fate.

Box 4 **Diagrammatic representation a gene regulatory network showing reprogramming potential involving activation and suppression switches that determine shoot and root fate during embryogenesis.**

**Figure:**
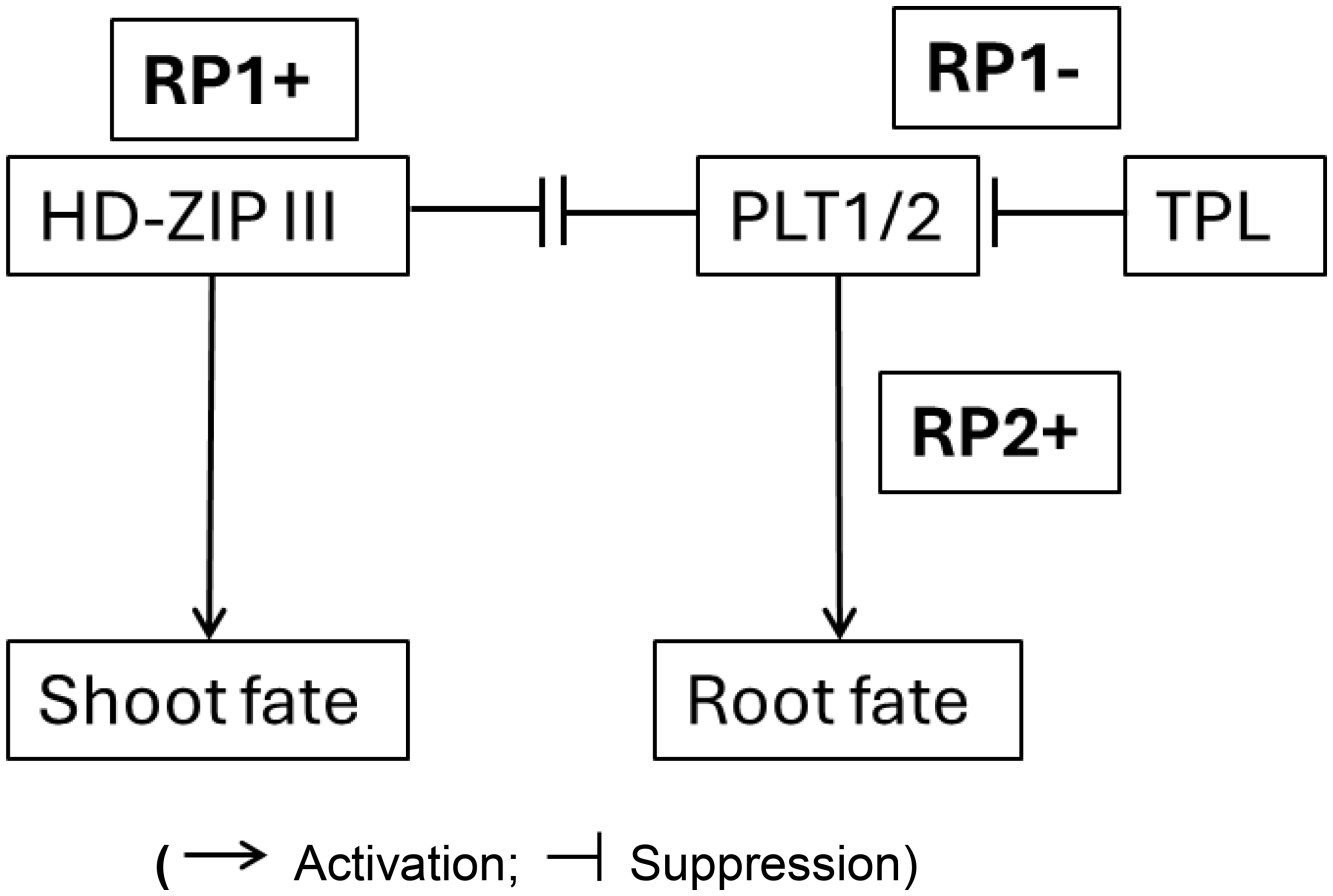

(Activation; Suppression)

The “-” state in our model represents an actively maintained suppressed developmental potential that can be readily reactivated (RP+) by environmental or internal cues, leading to the highly plastic and regenerative nature of plants.

Explanation of the Network:

• RP (Reprogramming Potential)

• HD-ZIP III promotes Shoot Fate

• HD-ZIP III represses PLT1/2; PLT1/2 represses HD-ZIP III

• TPL represses PLT1/2

• PLT1/2 promotes Root Fate

HD-ZIP III, when active, promotes shoot development. TPL normally keeps PLT1/2 in check. When TPL is dysfunctional (like in the *tpl-1* mutant), PLT1/2 become overly active, leading to the formation of roots even where shoots should be. There is an antagonistic relationship between PLT1/2 (root) and HD-ZIP III (shoot), which ensures the proper development of either root or shoot at each end of the embryo.

“Switch” denotes a molecular mechanism that can shift a cell or tissue from one developmental trajectory to another, or maintain a suppressed state that can be de-repressed. This is evident in the transition from an uncommitted state to either a shoot or root fate, or the maintenance of a basal domain that is prevented from adopting a shoot fate unless specific factors (like TPL) are mutated. For example, considering the temporal development of embryogenesis, the initial zygotic cell (or its early derivatives) has the potential for multiple fates, and the “switch” directs it towards a specific lineage (e.g., apical cell to embryonic, basal cell to suspensor, then further switches determining shoot vs. root poles).

“Reprogramming Potential” as the inherent capacity of plant cells/tissues to alter their developmental fate, a potential that is actively managed (activated or suppressed) during development. In the context of **Box 4** (GRN for shoot-root fate), RP1+ signifies the activation of the shoot program (e.g., via HD-ZIP III), while RP1- (linked to TPL activity) signifies the suppression of an alternative fate (e.g., TPL suppressing PLT1/2/root fate). The “reprogramming” aspect becomes evident when this balance is perturbed (e.g., *tpl* mutants where the apical region can be converted to a root), or during regeneration where a differentiated cell can give rise to a new organ by activating a suppressed embryonic-like program. Thus, the model describes not just the implementation of programs, but the underlying controlled potential for alternative fates and the mechanisms that ensure one fate prevails while others are suppressed yet potentially reactivable.

Ablation of the root meristem tip (130 µm from the Quiescent center [QC]) in a 5-day old seedling results in the regeneration of the meristem from surrounding cells in 72 hrs (Sena et al., 2009). The underlying molecular mechanism behind this regenerative replacement of ablated meristem was shown to evoke an embryo-like sequence of initiation of hypophyseal fate followed by a QC – columella cell fate and further resolution of the root apical meristem fate (Efroni et al., 2016). The positional information that initiates this embryo-like developmental sequence to replace the lost root meristem is provided by the interaction of auxin and cytokinin (Efroni et al., 2016). Auxin and cytokinin play critical roles during embryo development to establish the shoot/root/vascular stem cell programs, and their role in *in vitro* regeneration of plants is well established (Zhang et al., 2017; Ikeuchi et al., 2019; Dresselhaus and Jürgens, 2021). This corresponds to activation of the RP2+ embryonic program during root meristem ablation (**Figure 3**). While RP2+ directly refers to the activation of the primary root program during regeneration, the antagonistic suppression (the “-” state) might not always involve a shoot fate, but rather the suppression of other potential fates that could arise from the regenerating tissue if not directed towards a root.

More experimental evidence for the modular nature of the RP2+ program comes from a study that overexpresses the *PLETHORA* (*PLT*) pathway (Galinha et al., 2007), which is known to play an essential role in establishment of the embryonic root stem cell niche (Aida et al., 2004). Overexpression of *PLT2* resulted in an extended meristem size whereas removal of repression by the *RETINOBLASTOMA RELATED* (*RBR*), a tumor suppressor pathway, in the *PLT2* overexpressing line resulted in the assembly of two root meristems, one behind the other along the root axis (Galinha et al., 2007). This further demonstrates the modular nature of the embryonic RP2+ program in the activation of root meristem formation.

### Step 3: hypocotyl (shoot) – root fate

Late stage embryo development in Arabidopsis (bent stage) is characterized by the formation of the boundary zone between the hypocotyl and the radicle. This zone is referred to as the ‘collet’ and displays properties such as dense formation of root hairs in the seedling stage (Scheres et al., 1994; Sliwinska et al., 2012). The adventitious roots that develop in this region are referred to as anchor roots. Anchor roots can be induced to form by the excision of the radicle (primary root) tip and a recent study showed that a carotenoid derivative, anchorene, can induce the formation of anchor roots from pericycle cells via an auxin-independent pathway (Jia et al., 2019).

The collet region is referred to as the RP2- which can be activated to the RP3+ state to form specific adventitious roots called anchor roots. This reprogrammable state of the RP2- to RP3+ can be compared to the shoot-borne roots formed in monocot species in the early seedling phase, although the underlying mechanisms are different in origin, providing support for convergent evolution of shoot-borne roots (Orman-Ligeza et al., 2013). This shoot-borne root formation at post-embryonic phytomer nodal regions in monocots, referred to as crown roots (Gonin et al., 2019), can also be considered as an iteration of the RP3+ program with alternative molecular pathways such as the *LATERAL ORGAN BOUNDARIES* (*LBD*) gene family. A recent study in tomato identified a conserved superlocus encoded by the *LBD* that regulated above- and belowground root initiation (Omary et al., 2022). This LBD superlocus, which belongs to the subclass IIIA and IIIB, diversified very early in the evolution of angiosperms and initiates root development in several species through a common transition state. Thus, this LBD pathway might be operational in both the RP3+ and the RP4 programs related to root program activation (**Figure 3**).During the seedling phase, hypocotyl roots (HR; **Figure 2**) are initiated in Arabidopsis depending on the growth conditions (Verstraeten et al., 2014; Li et al., 2022; Zeng et al., 2022). The *LBD16/29* loci are involved in the initiation of these hypocotyl roots (Li et al., 2022), suggesting that post-embryonic root initiation on the embryonic hypocotyl falls under the RP3+ program (**Figure 3**).

### Step 4: patterning the embryonic “phytomer”

The bilateral symmetry of the cotyledonary primordia and the radial symmetry of the hypocotyl emerge from the differentiation of apical and basal domains of the embryo (Palovaara et al., 2016). We refer to this as an embryonic “phytomer” to highlight their similarity to the iterative post-embryonic phytomers, the bilateral symmetry of which are regulated by a similar gene regulatory network (GRN). When disrupted, polarity and symmetry determining factors (e.g., HD-ZIP III, KANADI (KAN) and YABBY) affect the polarity and patterning of the embryo and organs that are formed post-embryonically (Izhaki and Bowman, 2007; Bowman and Floyd, 2008). Loss of HD-ZIP III activity in the *phb phv rev* triple mutant results in loss of bilateral symmetry in the embryo and post-embryonic organs (Prigge et al., 2005). In the *KAN* triple mutant, *kan1 kan2 kan4*, HD-ZIP III activity spreads laterally due to the absence of inhibition by *KAN* gene activity. This results in the ectopic formation of leaf-like organs that are formed on the hypocotyl (Izhaki and Bowman, 2007). The contrasting effects of the HD-ZIP III and KAN activity is coordinated by auxin movement and signaling to specify bilateral symmetry (Izhaki and Bowman, 2007).

The specification of stem cells in the shoot meristem is due to the activity of a WUSCHEL/CLAVATA negative feedback loop along with cytokinin and KNOX gene family members, STM and BP (Aichinger et al., 2012). In the embryonic shoot meristem, WOX2 positively regulates HD-ZIP III activity that is required for shoot identity. WOX2 also regulates auxin pathway negatively and cytokinin pathway positively, to specify the shoot stem cell niche and bilateral symmetry of the cotyledons (Zhang et al., 2017). The role of auxin and cytokinin in the patterning of the apical domain of the embryo resembles the induction mechanism observed during plant regeneration (Zhang et al., 2017; Mathew and Prasad, 2021).

While the model describes the formation of bilaterally symmetrical cotyledons, a characteristic feature of eudicots like *Arabidopsis*, it is important to recognize that this specific morphology is modified in monocots. The “universal” nature of this step in the model does not lie in the final symmetrical output, but rather in the underlying genetic and developmental programs that are adapted to produce the single cotyledon (scutellum) seen in many monocots (Armenta-Medina et al., 2021). The proposed universal model hypothesis accommodates this divergence. The core concept for this stage is the patterning of the embryonic “phytomer,” which establishes the shoot’s foundational structures. In monocots, this same fundamental step of patterning occurs, but the developmental trajectory is altered, leading to a laterally positioned shoot meristem and the development of a single, highly specialized cotyledon. Therefore, the universality of this step is rooted in the establishment of an embryonic shoot program, with the specific symmetry of the cotyledons being a downstream modification that distinguishes eudicots and monocots.

The following two sections explore the reprogramming and regenerative programs (H2), focusing on the genes and GRNs that drive developmental shifts. These sections emphasize the crucial role of developmental switches involved in somatic and apomictic embryogenesis, activation of founder cells, and ectopic root formation that are also key players in embryonic development, specifically in cell fate specification, embryonic root initiation, and overall root development. This reinforces the interconnectedness of the two developmental programs - the shoot program (which operates iteratively post-embryonically) and the root program - within the context of embryonic development.

## The reprogramming potential with reference to plant embryogenesis (H2)

The ability of plant cells to form somatic embryos has been reported in several plant species from various tissues and the genetic mechanisms underlying this reprogramming event have been explored (Horstman et al., 2017a). Specifically, ectopic expression of the *AINTEGUMENTA-LIKE/PLETHORA* (*AIL/PLT*) clade gene *BABYBOOM*/*PLT4* can induce the formation of somatic embryos (Boutilier et al., 2002). The *BBM* genes (*BBM1, BBM2 and* BBM3) in rice have been shown to be critical to male transmitted pluripotency factors to initiate embryo development after fertilization; the triple *bbm1 bbm2 bbm3* mutant causes embryo arrest and abortion of the seeds (Khanday et al., 2019). A recent study in Arabidopsis shows that members of the AIL/PLT family, PLT1, PLT2, PLT3 and PLT4/BBM play an important role in the early initiation of embryogenesis and patterning based on: (1) the lethality of *plt2/bbm* double mutant; (2) the expression of *PLT2 and BBM* genes in the zygote and apical/basal cells after the first division during early embryogenesis, and; (3) the resemblance between the PLT regulome during early embryogenesis and that of meristematic cells (Kerstens et al., 2024). The redundant roles of the *PLT* genes in early embryogenesis and in the later specification of the root meristem activity suggests that the PLT genes were evolutionarily recruited for embryonic divisions and meristematic potential and later adapted for meristematic functions in the embryo of bipolar seed plants (Kerstens et al., 2024).

Another example of dual adaptation during seed evolution and reprogramming is observed in the functions of *LEAFY COTYLEDON* (*LEC*) gene family members. *LEC* transcription factors are master regulators of the seed maturation process during late embryogenesis which, when overexpressed during the vegetative phase, reprogram cells to induce somatic embryogenesis (Braybrook and Harada, 2008). Interestingly, *BBM*-induced somatic embryo formation involves the activation of the *LEC* TF- containing gene regulatory network (LEC1-ABI3-FUS3-LEC2 [LAFL]) (Horstman et al., 2017b). Recent studies suggest that the LAFL regulatory network is likely involved in phase transition during development via modulation of epigenetic pathways (Niu and He, 2019; Chen and Du, 2022). This highlights the central role of epigenetic mechanisms through which the master regulators like LEC and BBM reprogram cells towards totipotency (Peng et al., 2023).

The embryophyte genetic toolkit has repeatedly evolved to give rise to apomixis in both monocots and dicots (Bowman, 2022). Apomixis, a naturally occurring process of asexual reproduction through seeds whereby the embryo is a genetic clone of the mother plant, enables the preservation of a hybrid genotype over multiple generations and is found throughout the plant phylogeny. In the moss *Physcomitrium*, ectopic overexpression of the homeobox gene *BELL1* induces embryo formation and diploid sporophytes from specific gametophytic cells without fertilization (Horst et al., 2016). Naturally apomictic mechanisms in some angiosperms also appear to involve *BLH1* misexpression in the female gametophyte, reminiscent of the *eostre* mutant (Bezodis et al., 2022). In *Hieracium praealtum*, the *BLH1* orthologue is expressed in apomictic aposporous initial cells and early embryo sac cells (Okada et al., 2013). In *Arabidopsis*, ectopic expression under a germline-specific promoter of KNOX and BELL genes not normally expressed in the gametophytes both disrupts germ cell specification and causes defects in cell identity throughout gametophyte development – some mirroring events seen in natural apomicts (Bezodis et al., 2022). In *Pennisetum squamulatum*, multiple copies of BABY BOOM-like (BBM-like) genes that encode transcription factors previously implicated in somatic embryogenesis are found in the genetic locus associated with apomixis (Conner et al., 2015). The transfer of one of the BBM-like genes into pearl millet, rice and maize, is sufficient to trigger embryo formation without fertilization. Alternatively, the dandelion (*Taraxacum officinale*) *PARTHENOGENESIS* (*PAR*) gene encodes a protein with zinc finger and EAR domains (DNA binding and transcriptional repression) to activate embryo formation without fertilization (Wang and Underwood, 2023). Inducing apomixis in crops offers significant breeding potential by enabling the fixation of superior hybrid genotypes across generations through seeds. Transferring genes like *BBM-like* into crops such as rice and maize have successfully triggered embryo formation without fertilization, demonstrating a viable pathway to engineer this trait (Conner et al., 2017).

## The reprogramming potential of post-embryonic tissues (H2)

Plant cells are developmentally plastic with reference to their cell fate, as displayed by their regenerative capacity to form shoot/root meristems, somatic embryos and callus (Birnbaum and Alvarado, 2008). These regenerative potentials are predominantly activated in the pericycle-like cells in the shoot and the pericycle cells of the root in Arabidopsis, which led to the hypothesis that regeneration occurs via a root development pathway (Sugimoto et al., 2010). Subsequent studies demonstrate that genetic pathways regulating root meristem formation (*PLT1/PLT2*) during embryonic and post-embryonic stages of development are activated by *PLT3/PLT5/PLT7* during the formation of *de novo* root and shoot meristems from callus. However, in the case of shoot meristem specification, *PLT 3/5/7* activation is followed by activation of the shoot meristem pathway gene *CUC2* in a two-step process (Kareem et al., 2015). A recent study shows that the mechanical conflict created by differential cell wall loosening in callus progenitor cells which form shoot meristems versus surrounding cells is regulated by *CUC2* (Varapparambath et al., 2022). CUC2 activates the expression of *XTH9*, that encodes a cell wall loosening enzyme in cells surrounding the progenitor cells. This results in the localization of PIN1 auxin transport protein and the polarity protein SOSEKI2 in the progenitor cells, activating *de novo* shoot meristem formation (Varapparambath et al., 2022). Other pathways triggered by auxin and cytokinin involved in the specification of the shoot/root meristem specification (e.g., WOX and KNOX genes), and the LBD genes involved in lateral root/adventitious/*de novo* root formation have overlapping roles in the assembly of embryonic body plan in dicot and monocot species (Palovaara et al., 2016; Ikeuchi et al., 2019; Mathew and Prasad, 2021; Rashotte, 2021).

## Model summation

This “universal” model hypothesis of embryo development elucidates four steps that integrates the two hallmarks: (A) an iterative pattern of the phytomer (embryonic phytomer with apical meristem and iterative formation of post-embryonic phytomers by the SAM) and (B) the suppressed regenerative states that are reprogrammable (**Figure 3**). This model takes into account (1) the post-embryonic activity of the shoot apical meristem that iteratively form phytomers with axillary meristems; (2) the diverse outputs of the post-embryonic root types from root founder cells and collet zone (radicle/primary root; anchor root; shoot-borne root; adventitious root; lateral root; nodal root/crown root) (Dolan et al., 1993; Chandler, 2011; Gonin et al., 2019; Zhang et al., 2023) [**Box 5**]; and (3) the regenerative potential of plant tissues (e.g.,form shoot/root meristems, somatic embryos, callusing and wound healing). A diagrammatic representation of a GRN that functions as an activation – suppression switch is provided in **Box 4**. A detailed exploration of these GRNs that represent the activation - suppression switches will be the focus of an ensuing article.

Box 5 **Enhancing the universal embryo model - founder cells and the collet.**

The universal embryo model, while comprehensive, can be further enhanced by incorporating the concepts of root founder cells and the collet. Root founder cells, as the initiators of root formation, provide a modular perspective on root system development. These specialized cells give rise to the primary root (primary root founder cells; hypophyseal cell), lateral roots (lateral root founder cells; pericycle), and adventitious roots (adventitious root founder cells) (Dolan et al., 1993; Chandler, 2011; Gonin et al., 2019; Zhang et al., 2023). The collet, which forms the future root-shoot junction, plays a crucial role in integrating the development of the root and shoot systems, ensuring their proper connection and coordinated function.

Three distinct types of root founder cells, each with their specific WOX-ARF modules, play a crucial role in root initiation (Zhang et al., 2023):

1. **Primary root founder cells** (hypophysis in the embryo) utilize the **WOX9-ARF5** complex to activate RGF1 INSENSITIVES (RGIs), initiating primary root development.

2. **Lateral root founder cells** employ **ARF7/19** to activate RGIs and LBD16, initiating lateral root primordia without the involvement of intermediate-clade-*WUSCHEL-RELATED HOMEOBOX* (IC-WOX) (Liu and Xu, 2018).

3. **Adventitious root founder cells** in detached leaves utilize the **WOX11-ARF6/8** complex to activate RGIs and LATERAL ORGAN BOUNDARIES DOMAIN 16 (LBD16), initiating adventitious root primordia.

This division of labor among WOX-ARF modules (GRN) showcases a specialized mechanism for initiating different root types.

We hypothesize that the universal embryo model, with its foundation in the activation-suppression (RP1+ and RP1-) of the embryonic program, provides a framework for understanding root founder cell specification and the embedding of WOX-ARF modules.

Primary root founder cells

• **Location:** Embryo

• **WOX-ARF module:** WOX9-ARF5 complex

• **Activation:** RP2+ (activation of the root program)

• **Initiation:** Primary root development

Lateral root founder cells

• **Location:** Pericycle

• **WOX-ARF module:** ARF7/19 (without IC-WOX)

• **Activation:** RP5+ (activation of lateral root program)

• **Initiation:** Lateral root primordia

Adventitious root founder cells

• **Location:** Detached leaves

• **WOX-ARF module:** WOX11-ARF6/8 complex

• **Activation:** RP3+ (activation of adventitious root program)

• **Initiation:** Adventitious root primordia

This model effectively demonstrates how the universal embryo framework can be applied to understand the activation of different WOX-ARF modules in initiating distinct root types.

Hence, the **collet** plays a critical role in the **activation-suppression switch** of the universal plant embryonic model, particularly in the R2-, R3+.

• **RP2- (repressed shoot-borne root program):** The collet represents a developmental state where the shoot-borne root program is repressed. This suppression is crucial for maintaining the distinction between the shoot and root systems during embryogenesis.

• **RP3+ (activation of the shoot-borne root program):** The collet can transition to an R3+ state, activating the shoot-borne root program. This activation leads to the formation of adventitious roots, specifically the anchor roots. The ability of the collet to initiate anchor roots highlights its role in the developmental plasticity of the plant, allowing it to adapt to changing environmental conditions.

In summary, the collet’s involvement in the activation-suppression switch of the universal plant embryonic model demonstrates its crucial role in coordinating root development and facilitating the plant’s adaptation to its environment.

Founder cells of regeneration

Plant regeneration is the process by which plants can form new organs or tissues from detached or wounded parts. WOX11 is a key regulator of founder cell specification during regeneration (Wan et al., 2023). WOX11 is induced by auxin and wounding signals in regeneration-competent cells. It promotes the transition of these cells into founder cells for root or callus formation. WOX11 activates the expression of target genes that initiate root meristems, shoot meristem or callus primordia. The WOX11-mediated regeneration pathway is conserved in many plant species.

## Integrating Evo-Devo perspective of embryogenesis into the proposed universal model

The emergent ‘proto-meristems,’ which gave rise to shoot and root programs during the early evolution of land plants (as discussed in the Evolutionary origins section), represent foundational elements. These elements convergently and progressively integrated to form the bipolar embryonic body plan observed in extant lycophytes, monilophytes, gymnosperms, and angiosperms. The evolutionary origin and formation of meristems in different plant groups (**Figure 4**) provides a foundation for understanding the developmental and genetic pathways that gave rise to the “universal embryonic body plan” defined by dicot and monocot embryos (**Figures 2**, **3**). With the sequencing of genomes from across the land plant phylogeny (including early-diverging groups like bryophytes, lycophytes, and monilophytes, as well as later-diverging gymnosperms and angiosperms), comes a better understanding of the genetic and molecular mechanisms that underly embryonic and post-embryonic development in these different plant species. Looking within the angiosperms (dicots and monocots), an evolutionary perspective of the plant embryonic body plan provides a rich template to integrate the modular assembly of the shoot units during post-embryonic development (Hallmark 1) with the reprogramming potential as revealed by the regenerative pathways (Hallmark 2) into one framework. The proposed universal plant embryonic body plan that is distinct from the animal embryo models developed by this revised approach facilitates probing deeper into the genome of the land plants with reference to their genetics and adaptation. This model also offers a framework for interpretation and guides future research into the precise nature of the GRNs, further enabling the identification of both conserved and diversifying gene regulatory networks. The genetic mechanisms uncovered will, in turn, provide an enhanced ‘toolkit’ for understanding plant adaptation offering alternate blueprints for developing stress resilient crops that will be better adapted to future food production challenges in a changing global climate.
